# Moderate physical activity during late pregnancy enhances gut microbial network stability in pregnant women

**DOI:** 10.3389/fmicb.2025.1731350

**Published:** 2025-12-18

**Authors:** Kian Deng Tye, XiaoXia Ran, XiaoYi Liu

**Affiliations:** 1 Obstetrics Department, The Fifth Affiliated Hospital of Guangzhou Medical University, Guangzhou, Guangdong, China; 2 Neonatology Department, The First Affiliated Hospital of Jinan University, Guangzhou, Guangdong, China

**Keywords:** gut microbiota, host–microbe interaction, infection susceptibility, moderate physical activity, next-generation sequencing, pregnancy

## Abstract

**Aim:**

This study aimed to investigate the impact of moderate physical activity during late pregnancy on the overall structure and stability of the maternal gut microbiota, with particular emphasis on microbial network interactions and their potential implications for mucosal immune resilience.

**Methods:**

A prospective cohort study was initiated at 32 weeks of gestation, during which physical activity was assessed, and fecal samples were subsequently collected at full-term admission for delivery. Fecal samples were collected and analyzed using 16S rDNA sequencing, and daily physical activity levels were recorded. Participants were categorized into two groups based on the duration of moderate-intensity physical activity: T1 (≥30 min/day, 18 women) and T2 (<30 min/day, 5 women). Bioinformatics analyses were used to compare gut microbiota composition, diversity, and network interactions between the groups, and to assess correlations between microbial abundance and physical activity levels.

**Results:**

*Firmicutes*, *Bacteroidetes*, *Actinomycetes*, and *Proteobacteria* were the dominant phyla in both groups. Alpha diversity and principal coordinate analysis (PCoA) showed no significant differences in overall diversity. However, LEfSe analysis revealed an enrichment of *Christensenellaceae* and *Prevotella stercorea* in the T2 group. The gut microbial network in the T1 group was more complex and stable, with predominantly positive microbial correlations. Spearman analysis indicated significant associations between physical activity levels and specific gut microbes: sedentary behavior correlated negatively with *Romboutsia* (*p* = 0.033, *R* = −0.445) and was positively correlated with *Senegalimassilia* (*p* = 0.043, *R* = 0.443), light-intensity activity correlated negatively with *Phascolarctobacterium* (*p* = 0.015, *R* = −0.500), and moderate-intensity activity correlated positively with *Parasutterella* (*p* = 0.040, *R* = 0.432).

**Conclusion:**

Moderate physical activity during late pregnancy promotes a more stable and functionally interactive gut microbiota network. Such microbial resilience may strengthen mucosal immune regulation and reduce infection susceptibility during gestation. These findings highlight the potential of physical activity as a non-pharmacological strategy to modulate the maternal gut microbiome for improved host defense and health outcomes.

## Introduction

The adult intestine contains up to 100 trillion microbial cells, more than 10 times the total number of human cells in the body (approximately 30–40 trillion) (Dekaboruah et al., 2020). The total weight of the gut microbiota is estimated to be similar to the weight of a human brain, typically ranging from 1 to 2 kg in adults. Additionally, the gut microbiota is considered the second human genome, as metagenomic studies have revealed that the human gut contains 3.3 million distinct genes, which is 150 times more genes than the human genome (Zhu et al., 2010; Guevara-Cruz et al., 2019). An increasing number of studies have shown that alterations in the composition of the gut microbiota may lead to various chronic diseases, such as inflammatory autoimmune disorders, gut inflammation-related disorders, and cardiometabolic diseases, and specific, inflammatory bowel disease and diabetes (Durack and Lynch, 2019), to name a few. Regular physical exercise, as an important means of non-pharmacological treatment, can help prevent and delay the onset of disease while also providing various physical and psychological benefits, such as improved mood, reduced risk of behavior disorders, increased ability to perform daily activities, lower risk of falls, and improved social interactions for the patient (Vina et al., 2012; Hou et al., 2022). Besides, recent studies have reported that physical exercise can adjust the composition of the gut microbiota in healthy people, increase the number of beneficial bacteria, reduce the number of harmful bacteria, and positively impact human health through dialog between the gut microbiota and intestinal mucosal system (Clauss et al., 2021). The World Health Organization (WHO) recommends that all adults perform at least 150–300 min of moderate-intensity aerobic physical activity per week. For pregnant women, at least 150 min of moderate-intensity aerobic exercise per week is recommended (Kaiser and Campbell, 2014). Additionally, limiting the amount of sedentary time and replacing it with physical activity of any intensity (including light intensity) is beneficial to health (Bressa et al., 2017). According to joint recommendations from the American College of Obstetricians and Gynecologists (ACOG) and the American College of Sports Medicine (ACSM), pregnant women should engage in at least 30 min of moderate-intensity physical activity on most, if not all, days of the week (ACOG Committee Opinion No. 650: Physical Activity and Exercise During Pregnancy and the Postpartum Period, 2015). Moderate-intensity exercise (MIE) is usually defined as exercise that requires 3–6 metabolic equivalents (METs), a measure of the body’s energy expenditure during physical activity (Cerqueira et al., 2019; Huifen et al., 2022). During MIE, the exerciser can still have a normal conversation and have a 50–70% maximum heart rate but lower than 140 beats/min. Although ample evidence demonstrates the benefits of physical exercise for women during and after pregnancy, and even for neonates in the long term, the molecular mechanisms underlying the role of physical exercise during pregnancy remain elusive. Our previous studies have shown that probiotic supplementation can alter the placental microbiota composition and improve perinatal outcomes (Yang et al., 2020), and the change in placental microbiota can serve as the prognostic indicator (Chen et al., 2019). Moderate physical activity can modulate the composition and function of the gut microbiota and is also associated with increased production of short-chain fatty acids (SCFAs), which support immune function, improve nutrient utilization, and enhance gut barrier integrity through neural and hormonal regulatory pathways (Varghese et al., 2024). Physical activity can enhance the production of SCFAs in the gut, particularly acetate, propionate, and butyrate. These SCFAs not only provide energy for the host but also modulate the immune system and reduce inflammatory responses (Koh et al., 2016). Physical activity can strengthen the integrity of the gut barrier, reducing gut permeability and preventing the entry of harmful substances into the bloodstream. This helps maintain the stability of the gut microbiota and the health of the host. In pregnant women, the impact of physical activity on gut microbiota may be even more pronounced. Pregnancy is a unique physiological state, and changes in physical activity levels can affect the metabolic health of the pregnant woman and the development of the fetus through the aforementioned molecular pathways (Soma-Pillay et al., 2016). Despite increasing evidence linking physical activity to gut microbiota composition, very few studies have examined how different durations of moderate-intensity physical activity in late pregnancy influence microbial network structure and stability key ecological features that underpin maternal immune resilience. This study uniquely integrates microbial diversity, network topology, and activity-related taxa to provide novel insights into how late-pregnancy physical activity modulates the gut ecosystem.

## Materials and methods

This research project was approved by the Evaluation Committee of Human Body Research Institutions (IRB) of the First Affiliated Hospital of Jinan University (Approval No. 2019-011). All participants provided written informed consent prior to enrollment (Supplementary material 1). The study was conducted in accordance with the ethical principles outlined in the Declaration of Helsinki.

### Inclusion and exclusion criteria

A total of 23 healthy pregnant women were recruited at the First Affiliated Hospital of Jinan University between January 2018 and January 2019. Physical activity information and informed consent were obtained at 32 weeks of gestation. Fecal samples were collected later during full-term admission (≥37 weeks) under sterile conditions. This sampling period allowed us to relate late-pregnancy physical activity patterns to gut microbiota composition at term. The inclusion criteria for this study were healthy pregnant women aged between 20 and 35 years old, with primigravida, single pregnancy, and Chinese nationality. The exclusion criteria included gastrointestinal diseases or any medical, surgical, and obstetric complications, antibiotic use during pregnancy, and obesity (BMI (kg/m^2^) ≥ 30). Informed consent was signed at 32 weeks of pregnancy. According to the duration of moderate-intensity physical activity, these women were divided into the T1 (time > 30 min, 18 women) and T2 groups (time < 30 min, 5 women).

### Pregnancy physical activity questionnaire

The established pregnancy physical activity questionnaire (PPAQ) was used to evaluate the parameters of physical activity in this study (Chasan-Taber et al., 2004). When the participant was admitted to the hospital for delivery, the PPAQ questionnaire was administered. The completion rate of the questionnaire was 100%. Metabolic equivalents (METs) were used as the objective measure to measure the intensity of physical exercise relative to their resting metabolic rate (Jetté et al., 1990; Zientara et al., 2021; Jaeger et al., 2022). Physical activities were classified into different categories based on intensity levels, including sedentary (<1.5 METs), light intensity (1.5 < 3.0 METs), moderate intensity (3.0–6.0 METs), or vigorous intensity (>6.0 METs) (Table 1). However, in practice, since some participants were unable to accurately provide the MET values of their activities, we ultimately adopted the type and duration of activities, which are easier to obtain and can be accurately recalled by participants, as an alternative standard. According to the WHO recommendation, it is recommended that the duration of moderate-intensity physical activity performed by pregnant women should not be less than 150 min per week (Peterson et al., 2002; Khairnar and Parija, 2007). To facilitate the data collection and statistical analysis, we used 30 min of moderate-intensity physical activity per day as the standard for grouping.

**Table 1:** Physical activity intensity.

Total samples (*n* = 23)	METs.hour (mean ± SD)
Sedentary (<1.5 METs)	5.31 ± 2.20
Light (1.5 < 3.0 METs)	22.64 ± 5.02
Moderate (3.0–6.0 METs)	4.63 ± 4.14

### Sample collection

In this study, the sampling was conducted during the admission of pregnant women at full-term pregnancy, which specifically includes fecal samples and questionnaires. Fresh fecal of pregnant women were collected and standardized under strict aseptic conditions. Three to 5 g of fecal were weighed, placed in a sterile specimen box, and stored in a − 80 °C freezer until further use.

### DNA extraction and 16S rRNA V4 region amplicon preparation

Total genomic DNA was extracted from fresh fecal samples using a modified cetyltrimethylammonium bromide (CTAB) protocol optimized for complex microbial communities. Briefly, approximately 1.0–1.5 g of homogenized fecal material was transferred to a pre-chilled tube and thoroughly ground using a sterile mortar and pestle or mechanical homogenizer in the presence of liquid nitrogen or dry ice to ensure complete cell disruption. The homogenized material was mixed with pre-warmed (65 °C) 2% CTAB extraction buffer and gently inverted to allow efficient lysis. Samples were incubated at 65 °C for 30–60 min with intermittent mixing to facilitate release of genomic DNA. Following lysis, an equal volume of phenol:chloroform:isoamyl alcohol (25:24:1) was added, and the mixture was vortexed and centrifuged to separate phases. The aqueous phase containing nucleic acids was recovered and subjected to an additional extraction using chloroform:isoamyl alcohol (24:1) to remove residual proteins and contaminants. DNA was precipitated by adding cold absolute ethanol or isopropanol in equal volume, gently inverting, and incubating at −20 °C for 30 min. The precipitated DNA was collected by centrifugation and washed with 75% ethanol to remove remaining salts and CTAB. After air-drying, DNA pellets were resuspended in nuclease-free water and stored at −20 °C until downstream analysis.

The 16S rRNA genes of the V4 region were amplified using the primers 515F-806R (Deissová et al., 2023), with each PCR reaction containing 1 μL of each primer, 1–2 μL of genomic DNA template (approximately 10–20 ng), 12.5 μL of Phusion® High-Fidelity PCR Master Mix (New England Biolabs), and nuclease-free water added to a final volume of 25 μL. The PCR products were assessed by electrophoresis on a 2% agarose gel with 1× loading buffer (containing SYB green). After detection, PCR products with similar concentrations were pooled and purified through 1× tris acetate-EDTA and 2% agarose gel electrophoresis. The purified mixture of PCR products underwent further purification using the Qiagen Gel Extraction Kit (Qiagen, Germany). Subsequently, the Illumina TruSeq®DNA PCR-Free Sample Preparation Kit was utilized for library construction. The library quality was evaluated using the Qubit® 2.0 Fluorometer (Thermo Scientific, USA) and the Agilent Bioanalyzer 2,100 system (Agilent Technologies, USA). Obesity during pregnancy raw data were initially processed using the RS_ReadsOfinsert.1 protocol within SMRT Portal version 2.3.0 for barcode demultiplexing, followed by chimera filtering to obtain clean data suitable for subsequent analysis. In the subsequent USEARCH analysis, the clustering parameter for sequence identity was set to 97%. All the filtered raw data were then re-mapped to OTU representative sequences to generate an OTU abundance table. The original OTU table was created using custom scripts and was statistically analyzed at different taxonomic levels. To cluster OTUs, the data were subjected to read joining and filtering on the HiSeq PE250 sequencing platform.

### Microbial analysis and statistical analysis

The NovoMagic is a cloud-based platform developed by Novogene. We used the novoMagic platform to analyze microbial diversity, principal coordinate analysis (PCoA), linear discriminant analysis effect size (LEfSe), and Spearman analysis. Regarding the microbial diversity analysis, alpha diversity reflects the complexity of species diversity for a sample, and indices, including the Shannon and Simpson indices, were used for analysis. In contrast to alpha diversity, beta diversity describes diversity between samples, namely, the similarity between two samples, and it can be used to evaluate the distance between samples. The distance matrices served as inputs for PCoA, which represent the phylogenetic distances between samples. The beta diversities of the two different groups were calculated and visualized through different dimensional PCoA analyses using the weighted UniFrac distances of the 16S rRNA gene between microbial communities. Co-occurrence networks were constructed using the Spearman analysis and visualized by Gephi software (Version 0.9.2). To avoid the interference of rare or polluting species on the results, we only reserved the OTUs that existed in more than 50 percent of the samples to construct the network. Moreover, we calculated the topological properties to evaluate the characteristics of the network. Excel was used for data entry and calculation. Descriptive data were calculated and expressed as the mean ± standard deviation. The significance of baseline characteristics and topological properties were tested by the Wilcoxon or Student’s test. *p* value < 0.05 was considered statistically significant.

## Results

### The parameter of physical activity of pregnant women

As shown in Tables 2, 23 women in total had conducted moderate-intensity activity during the test period, and they were separated into two groups according to their activity durations. Eighteen women (78.26%) had conducted moderate-intensity activity for more than 30 min, while 5 women (21.74%) had conducted moderate-intensity activity for less than 30 min.

**Table 2:** Moderate-intensity activity time.

Moderate-intensity activity time	*N*	Ratio (%)
≥30 min (group T1)	18	78.26%
<30 min (group T2)	5	21.74%

### Effect of physical activity on the gut microbiota of pregnant women

**Figure 1:**
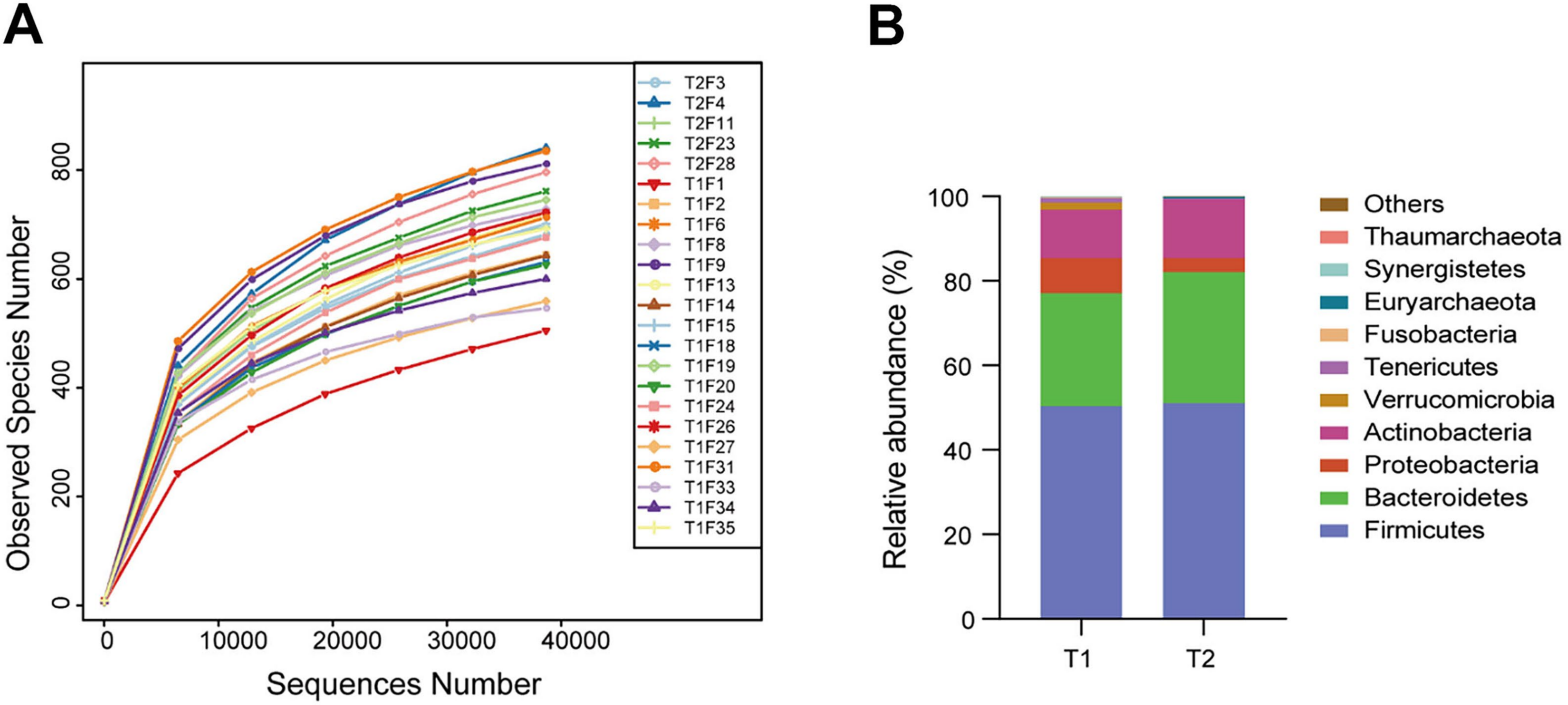
Dilution curve of gut microbiota of each sample and top 10 species of two groups at the phylum level (T1 group: time >30 min, T2 group: time <30 min). **(A)** Dilution (Rarefaction) Curves of Gut Microbiota. **(B)** Composition of Dominant Gut Microbial Phyla.

A certain amount of sequencing data was randomly selected from each sample, and the number of representative species was counted. The dilution curve was constructed based on the extracted sequencing data and the corresponding species number. As the curve shows, the number of OTUs in all samples increased with increasing sequencing quantity and finally plateaued. This result shows that the sequencing quantity was enough to achieve the ideal sequencing depth (Figure 1A).

**Figure 2:**
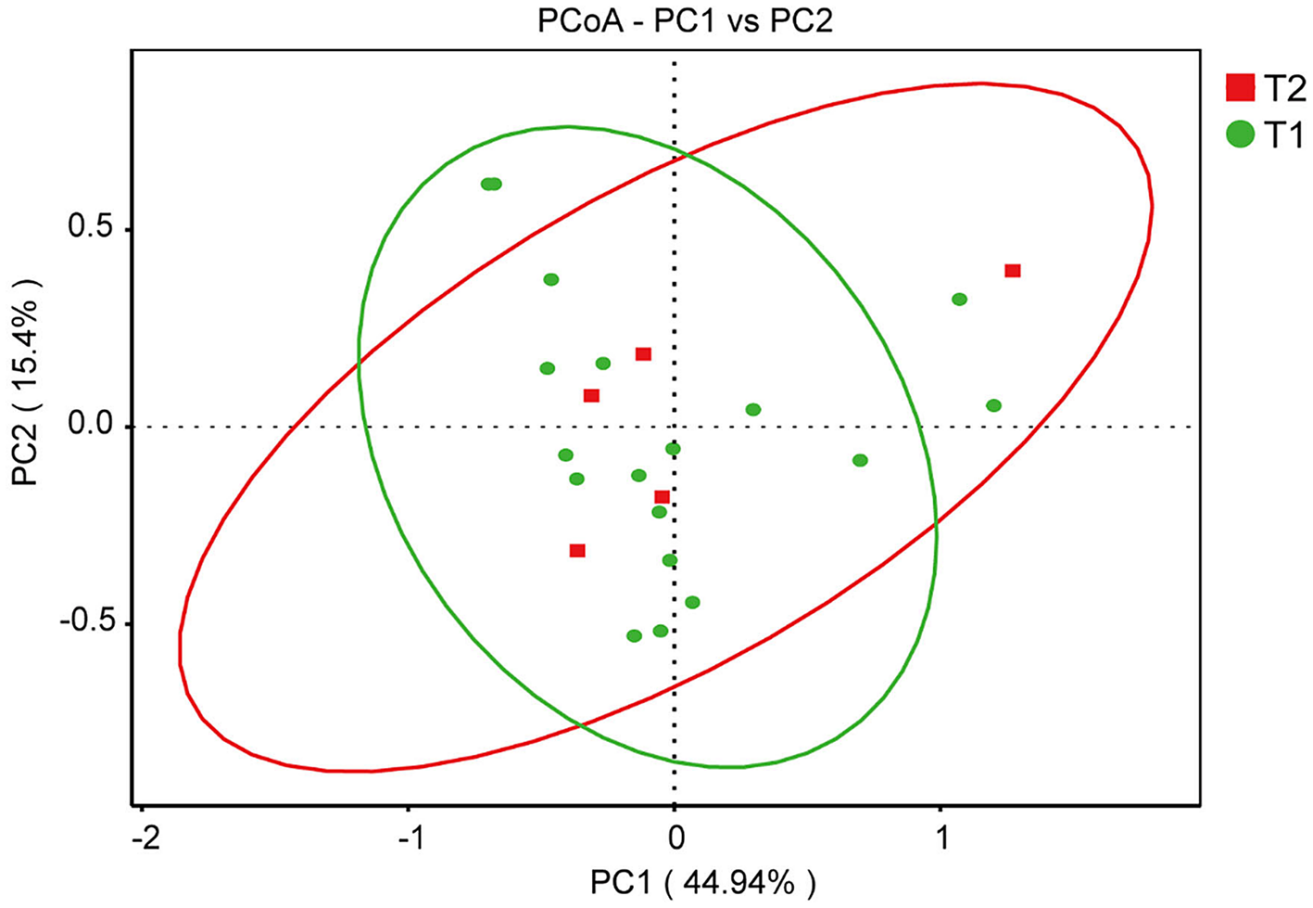
The PCoA analysis of gut microbiota both T1 group and T2 group.

Phyla and genera were annotated for the top 10 species at the taxonomic level (Figure 1B). The top four most abundant microbiota constituents at the phylum level in all the fecal samples are *Firmicutes*, *Bacteroidetes*, *Actinobacteriota* and *Proteobacteria*. In group T1, the top four bacterial phyla are *Firmicutes* (50.49%), *Bacteroidetes* (26.60%), *Actinobacteriota* (11.43%) and *Proteobacteria* (8.25%). In group T2, the top four bacterial phyla were *Firmicutes* (51.07%), *Bacteroidetes* (30.94%), *Actinobacteriota* (13.93%) and *Proteobacteria* (3.35%). The results of the Principal Coordinates Analysis (PCoA) show that the components TI and T2 are similar, indicating that exercise does not cause changes in the microbiota (Figure 2).

### Co-occurrence network analysis

**Figure 3:**
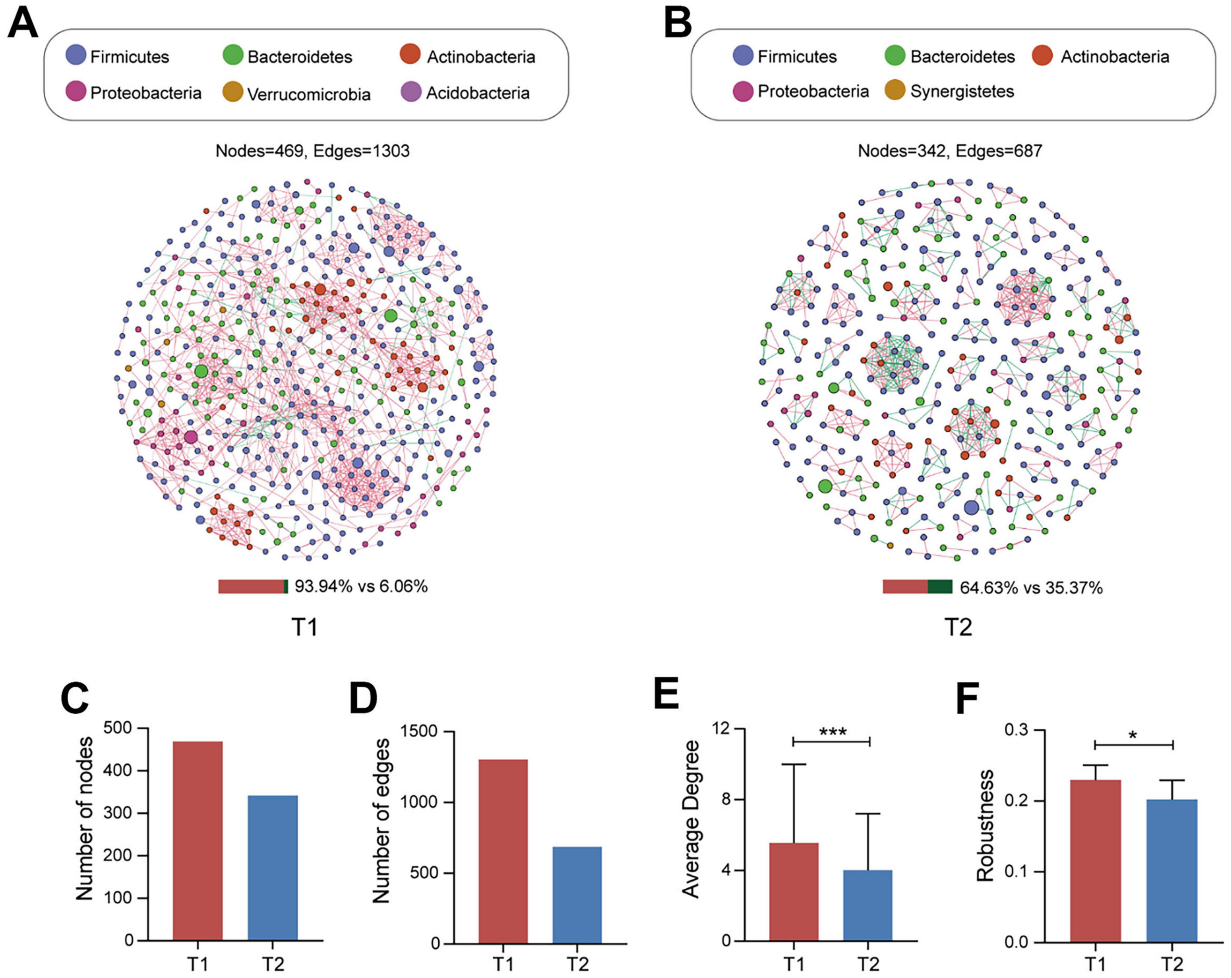
Co-occurrence networks and topological properties of the T1 group and the T2 group. **(A,B)** Intestinal microbial networks for the T1 group and the T2 group, respectively. Each node represents one OTU; different colors represent different phyla. Edges indicate significant correlations between OTUs. The red edge indicates that the nodes are positively correlated, whereas the green edge indicates that the nodes are negatively correlated. The bar charts below the networks represent the ratio of positive and negative correlations. **(C)** Total nodes of network. **(D)** Total links of network. **(E)** Average degree of network. **(F)** Robustness of network.

As microbes do not exist independently, and their interactions play an important role in maintaining host health, given that no significant differences were observed between participants, we further constructed gut microbiota networks for the T1 and T2 groups (Figures 3A,B). The gut microbiota network of the T1 group had more nodes, edges and positive correlations (Figures 3C,D). Moreover, the average degree, an indicator of network complexity, was higher in the T1 group (*p* < 0.05) (Figure 3E). Additionally, robustness, which represents the network stability, was also higher in the T1 group (*p* < 0.05) (Figure 3F). These results suggested that pregnant women with moderate exercise intensity greater than 30 min a day have more complex and stable gut microbiota networks.

### Analysis of differentially abundant species among the gut microflora

**Figure 4:**
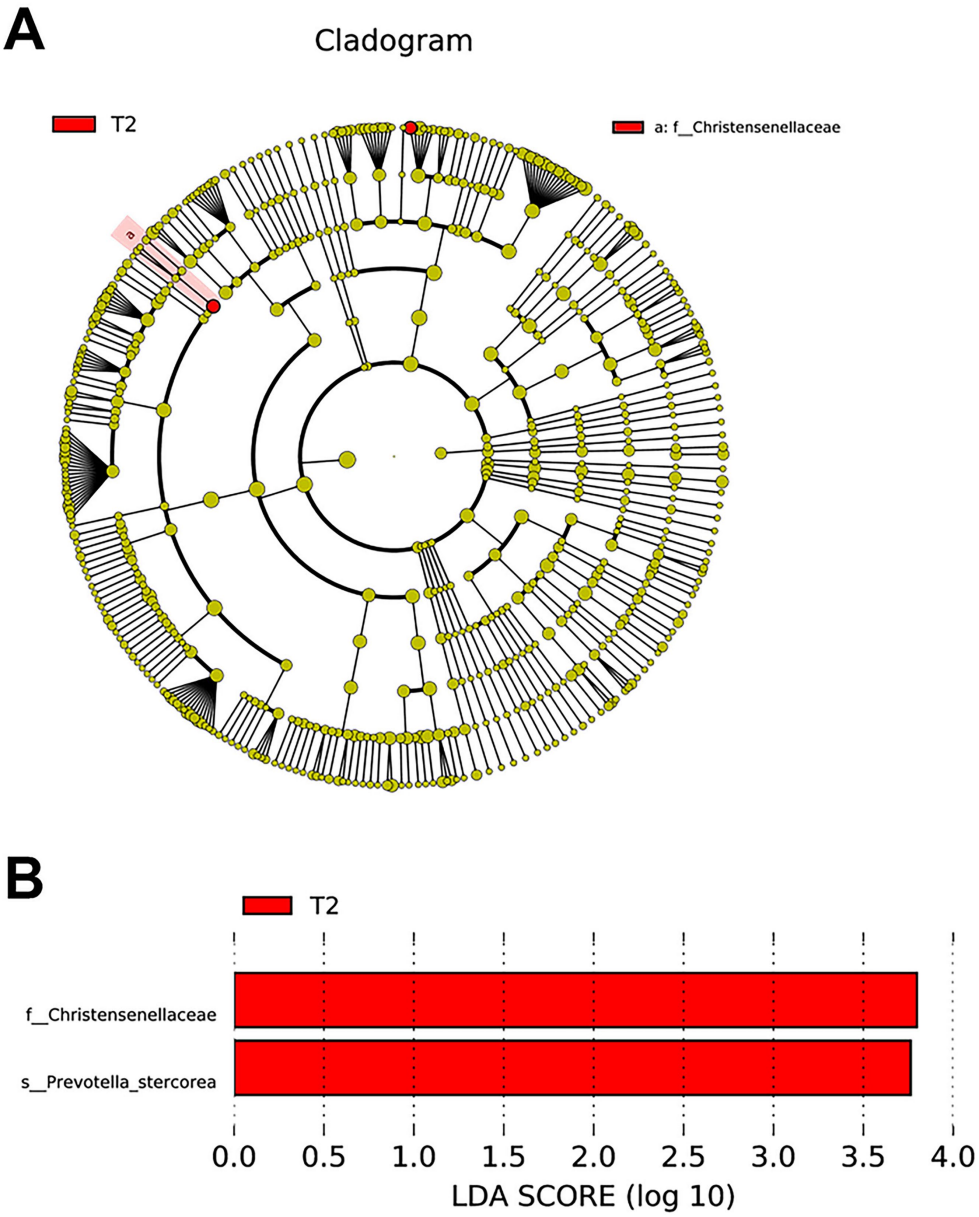
Distinct species between T1 and T2 groups. **(A)** Evolutionary branch diagram. **(B)** LEfSe analysis compared all taxonomy between groups. Identified two biomarkers when LDA score = 3.

The nonparametric factorial Kruskal–Wallis (KW) rank-sum test was used to detect features with significantly differential abundance, and LDA was used to estimate the effect size of each differentially abundant feature. The analysis showed that the family *Christensenellaceae* and species *Prevotella stercorea* were enriched in the T2 group when the LDA value was 3 (Figure 4).

**Figure 5:**
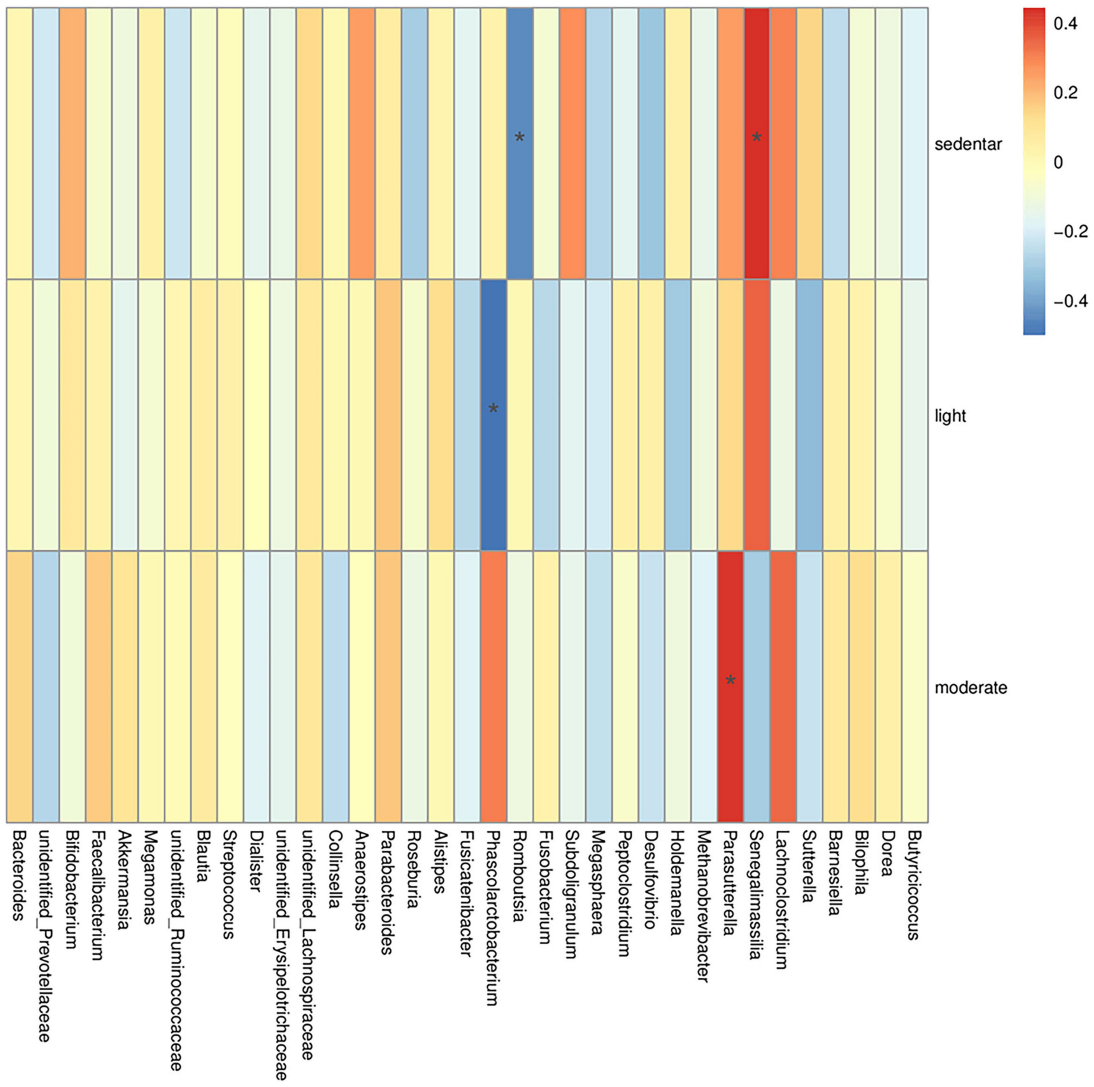
Heatmap of Spearman’s rank correlations between differentially abundant genera and physical activity. Blue represents a negative correlation, red represents a positive correlation, and the darker the color, the more significant the correlation. We observed that sedentary lifestyle (<1.5 METs) was negatively correlated with *Romboutsia* (*R* = −0.445, *p* = 0.033) and positively correlated with *Senegalimassilia* (*R* = 0.443, *p* = 0.034). Light-intensity physical activity was negatively correlated with *Phascolarctobacterium* (*R* = −0.500, *p* = 0.015). Moderate-intensity physical activity was positively correlated with *Parasutterella* (*R* = 0.432, *p* = 0.040).

Spearman correlation analysis was performed to evaluate the associations between differentially abundant gut microbial genera and physical activity intensity. To explore the correlation between the intestinal microbiota in pregnant women and different physical activity intensities, the Spearman correlation was further used to identify the main affected bacteria. A heatmap of Spearman’s rank correlations between differentially abundant genera and differentially abundant pathways is shown in Figure 5. We observed that a sedentary lifestyle (<1.5 METs) was negatively correlated with *Romboutsia* (*R* = −0.445, *p* = 0.033) and positively correlated with *Senegalimassilia* (*R* = 0.443, *p* = 0.034). Light-intensity physical activity was negatively correlated with *Phascolarctobacterium* (*R* = −0.500, *p* = 0.015). Moderate-intensity physical activity was positively correlated with *Pasteurella* (*R* = 0.432, *p* = 0.040). All the correlations were significant (Table 3).

**Table 3:** Spearman’s rank correlations.

Impact factor	Eubacterium	Correlation	*P*
Sedentary	*Romboutsia*	−0.445	0.033^*^
	*Senegalimassilia*	0.443	0.034^*^
Light	*Phascolarctobacterium*	−0.500	0.015^*^
Moderate	*Parasutterella*	0.432	0.040^*^

*Denotes a statistically significant correlation (*P* < 0.05) in Spearman’s rank analysis.

## Discussion

With the improvement in people’s living standards and changes in eating habits, many women have developed obesity during pregnancy. Obesity during pregnancy not only increases the risk of maternal complications but also negatively affects fetal growth and development. Recent studies have shown that pregnancy affects women’s metabolism and that physical activity could effectively prevent obesity in pregnant women (Evans et al., 2014; Kang et al., 2014; Campbell et al., 2016). For pregnant women, particularly those with specific medical or musculoskeletal concerns, high-intensity exercise may increase the risk of injury or adverse pregnancy outcomes. Therefore, the ACOG and ACSM recommend that pregnant women engage in at least 30 min of moderate-intensity physical activity on most days of the week. A total of 78.26% of pregnant women in our study met the criteria. This finding suggests that we should pay more attention to the necessary guidance for pregnant women in future clinical work and increase their participation in moderate-intensity activities and a balanced diet. A major refinement in this Discussion is the shift in emphasis toward our central finding: women engaging in >30 min of moderate-intensity activity exhibited a more coherent and resilient gut microbial network structure. It should be noted that the classification of participants was based specifically on moderate-intensity activity performed during late pregnancy, as assessed by the PPAQ. Physical activity earlier in pregnancy was not separately quantified. While long-term lifestyle habits may contribute subtly to baseline microbiota structure, the absence of significant differences in alpha and beta diversity between groups, combined with marked differences in microbial network stability, suggests that late-pregnancy activity patterns were the predominant driver of the observed microbial interaction changes. The absence of significant differences in alpha or beta diversity combined with clear differences in interaction networks suggests that late-pregnancy activity patterns were the primary driver of microbial structural changes observed in this study. As an intervention method, exercise can effectively regulate the distribution of the gut microbiota, improve the diversity of the flora, increase the number of beneficial bacteria, promote the balance of the intestinal microecology, and improve the health of the body (Mailing et al., 2019; Mohr et al., 2020; Clauss et al., 2021; Joos et al., 2025). However, our findings demonstrate that network-level characteristics rather than community composition are particularly responsive to moderate physical activity during pregnancy. Studies have shown that exercise regulates metabolism and the gut microbiota (Canani et al., 2011; Tilg and Moschen, 2014; Cronin et al., 2018). Proper exercise can promote the rhythm of the digestive system and maintain intestinal homeostasis. Clarke et al. (2014) found that rugby players’ gut microbiota diversity was higher than nonathletes. The increase in gut microbiota diversity during exercise may indicate a healthier intestinal environment, but the relationship between gut microbiota diversity and exercise intensity is still unclear. Denou et al. (2016) showed that high-intensity intermittent exercise increased the alpha diversity and the proportions of distal intestinal and fecal microbes during diet-induced obesity, especially the abundance of Bacteroides in the colon and cecum. In this study, after high-throughput sequencing of fecal samples from pregnant women, we observed that both the T1 and T2 groups displayed a similar and balanced distribution of the major phyla (*Firmicutes*, *Bacteroidetes*, *Actinobacteriota*, *and Proteobacteria*), and there were no significant differences in alpha diversity indices between the two groups. This indicates that overall richness and taxonomic composition remain stable, and that the beneficial effect of exercise manifests primarily through enhanced network stability and cooperative microbial interactions. These findings indicate that overall phylum-level balance is not driven by activity duration. Instead, the beneficial effect of exercise observed in this study appears primarily in the enhanced microbial network stability rather than in broad compositional shifts. Although alpha-diversity indices did not differ significantly between groups, this suggests that overall microbial richness was not strongly influenced by the exact duration of moderate activity, provided that participants maintained regular daily activity. Differences between groups became apparent only in beta-diversity and network structure, indicating that microbial community interactions not richness were more sensitive to variations in activity duration. Although both groups showed similar phylum-level distributions, the enhanced network stability and cooperative interactions in the T1 group indicate that regular moderate exercise supports a more functionally homeostatic microbiota, which may contribute to reduced infection susceptibility. Their associations with metabolic or inflammatory conditions reported in previous studies suggest potential links between moderate activity and a healthier metabolic-microbial milieu, although these relationships require further mechanistic investigation. Firmicutes is one of the most abundant bacterial phyla in the human gut and is closely related to the host’s metabolic functions. They play important roles in energy acquisition, the synthesis of short-chain fatty acids, and the regulation of the host’s immune system. Moreover, changes in the abundance of Firmicutes are closely associated with the development of metabolic diseases such as obesity and diabetes (Boggio Marzet et al., 2022). Bacteroidetes is another important gut bacterial phylum, renowned for its ability to degrade complex carbohydrates (such as dietary fiber). They produce short-chain fatty acids in the gut, providing energy for the host and participating in the regulation of gut barrier function and immune responses (Maslowski and Mackay, 2011). Although *Actinobacteria* are relatively less abundant in the gut microbiota, their members particularly *Bifidobacterium* play important roles in maintaining gut microbial balance and promoting host health. *Bifidobacterium* can inhibit the growth of harmful bacteria and strengthen gut barrier function, partly through the production of acetate (Fukuda et al., 2011). *Proteobacteria* contains many potentially pathogenic bacteria, but under normal conditions, they also play important roles in the gut microbiota. Changes in the abundance of *Proteobacteria* are associated with gut inflammation and the occurrence of certain diseases, with functions mainly involving the metabolism of nutrients and the regulation of the host’s immune system (Hou et al., 2022). In summary, these four phyla have extensive physiological functions in the gut microbiota, influencing the host’s health status through complex interactions. Future research can further explore the specific mechanisms of action of these phyla under different physiological and pathological conditions, providing new ideas for disease prevention and treatment. In this context, the balanced abundance of these phyla in active pregnant women supports the idea that exercise promotes a homeostatic microbiota composition capable of mitigating infection susceptibility.

Since there was no significant difference in gut microbiota composition and diversity among pregnant women, functional and structural differences in microbial networks, we further performed microbial networks and LEfSe analysis. In this study, pregnant women who exercised at a moderate intensity for more than 30 min had a more complex and stable gut microbiota network. Moreover, they had a higher positive correlation between gut microbes, suggesting that most gut microbes are cooperative. Cooperation may be the main association between microbes in a relatively healthy intestine (Cong et al., 2019). Additionally, we found that two different microbial taxa displayed significant abundance differences (LDA score > 3) between the T1 and T2 groups. The LEfSe analytic approach, found that *Christensenellaceae* and *Prevotella stercorea* were more abundant in the T2 group than in the T1 group. Some studies have found that the family *Christensenellaceae* positively correlates with blood pressure (Bier et al., 2018). However, *Prevotella stercorea* was more abundant in patients with polycystic ovary syndrome (PCOS) (Dong et al., 2021). These findings indicate that the T1 group of pregnant women may have a healthier flora structure than the T2 group of pregnant women, further indicating that physical activity for more than 30 min is favorable for the gut microbiota of pregnant women. To explore potential clinical paths that change the maternal gut microbiota, we investigated whether differentially abundant taxa were associated with physical activity intensity indices in pregnancy. This study found some significantly differentially abundant genera related to physical activity intensity, such as *Romboutsia*, *Senegalimassilia, Phascolarctobacterium,* and *Parasutterella*. Recent studies have found that the Romboutsia genus numbers were decreased in a Crohn’s disease group compared with those in a healthy subject group (Qiu et al., 2020). Girls with PCOS had a lower abundance of the genus *Senegalimassilia* than controls (Garcia-Beltran et al., 2021). Correlation analysis illustrated that the abundance of the gestational diabetes mellitus (GDM)-enriched genus *Parasutterella* was negatively correlated with fasting blood glucose levels (Ma et al., 2020). The positive correlation between moderate physical activity and *Parasutterella* abundance observed in our cohort aligns with reports of reduced fasting glucose and lower GDM risk associated with this genus (Ma et al., 2020), suggesting a potentially beneficial metabolic and immune-modulatory profile in physically active pregnant women. Beyond individual taxa, the enhanced microbial network stability in the T1 group may contribute to greater functional redundancy and resilience, features known to support mucosal immune homeostasis by reducing barrier disruption and endotoxin translocation (Johnson et al., 2020). Additionally, moderate physical activity (Supplementary material 2) is associated with increased production of short-chain fatty acids, including acetate, propionate, and butyrate, which exert well-characterized immunoregulatory effects through activation of G-protein–coupled receptors (GPR41/43), modulation of Treg responses, and reinforcement of epithelial tight junctions (Supplementary material 3). These mechanisms provide a plausible biological framework linking the more coherent microbial interaction networks in active pregnant women with enhanced host immune resilience (Furusawa et al., 2013). In our study, we observed significant differences in the abundance of *Senegalimassilia* and *Phascolarctobacterium* between the two groups of samples. Combining our findings with previous research, we believe that these differences may be associated with the health status of the host. For example, changes in the abundance of *Phascolarctobacterium* may be related to the occurrence of metabolic diseases, while changes in *Senegalimassilia* may reflect the dynamic shifts in the gut microbiota. It is important to note that individual taxa may not consistently reflect beneficial or adverse physiological states across different populations. Accordingly, our interpretation focuses on the broader ecological effect of moderate physical activity, particularly the enhancement of microbial network complexity and stability, which represents a more integrative indicator of gut ecosystem resilience. Thus, even when certain genera increase in sedentary individuals, the overall microbial interaction network remains more robust in the physically active group (Bucher-Johannessen et al., 2024). The above results show that moderate-intensity physical activity can reduce the incidence of gestational diabetes. Future research can further explore the specific mechanisms of action of these genera under different disease conditions, providing new insights for disease prevention and treatment.

With the advancement of high-throughput sequencing, people have increasingly understood the gut microbiota. Presently, research on the gut microbiota focuses on the correlation between disease occurrence and development, while research on the gut microbiota of pregnant women is rare. In short, the effect of physical activities in pregnancy on the gut microbiota may increase gut microbiota barrier function; accelerate endotoxin excretion; increase the amount of short-chain fatty acids produced by beneficial bacteria; reduce pathogen abundance, inflammatory factor levels, and oxidative stress; and promote intestinal homeostasis balance (Hughes and Holscher, 2021). This study was limited by its small sample size. In addition, baseline microbiome characteristics before pregnancy or in early gestation were not collected, and detailed dietary intake, probiotic consumption, and gestational weight gain data were not systematically recorded. The absence of these variables limits our ability to fully distinguish exercise-related effects from natural physiological changes during pregnancy. Although strict exclusion criteria were applied to reduce major confounders, future prospective studies with controlled dietary and lifestyle factors are needed to strengthen causal inference. Nevertheless, we observed that exercise had a significant effect on the gut microbiota network of pregnant women. Large-scale rigorous randomized controlled trials are needed to ensure the generalizability of the data.

## Conclusion

This study suggests that moderate, sustained physical activity during late pregnancy is associated with a more stable and cooperative gut microbiota network, even in the absence of major changes in overall microbial diversity. Such improvements in microbial network structure may support mucosal barrier function and contribute to more balanced immune signaling. Although individual taxonomic shifts were modest, the overall pattern indicates that regular moderate-intensity exercise—as recommended by WHO and ACOG—may serve as a safe, non-pharmacological strategy to support maternal gut microbial resilience. Given the small sample size and the absence of early-pregnancy baseline microbiome or dietary data, these findings should be interpreted cautiously. Future longitudinal studies incorporating metagenomic, metabolomic, and immune profiling approaches are needed to clarify the mechanistic pathways linking physical activity, microbial network dynamics, and maternal–fetal health outcomes.

## Data Availability

The original contributions presented in the study are included in the article/Supplementary material, further inquiries can be directed to the corresponding author/s.
