# Supplementary material for: Moderate physical activity during late pregnancy enhances gut microbial network stability in pregnant women
Source: Front Microbiol. 2025 Dec 18;16:1731350. doi: 10.3389/fmicb.2025.1731350 (PMC12756603; doi:10.3389/fmicb.2025.1731350)
Supplement: Supplementary file 1 [file Data_Sheet_1.docx]

Supplementary material

Supplementary File 1: Moderate Physical Activity Assessment Form

**
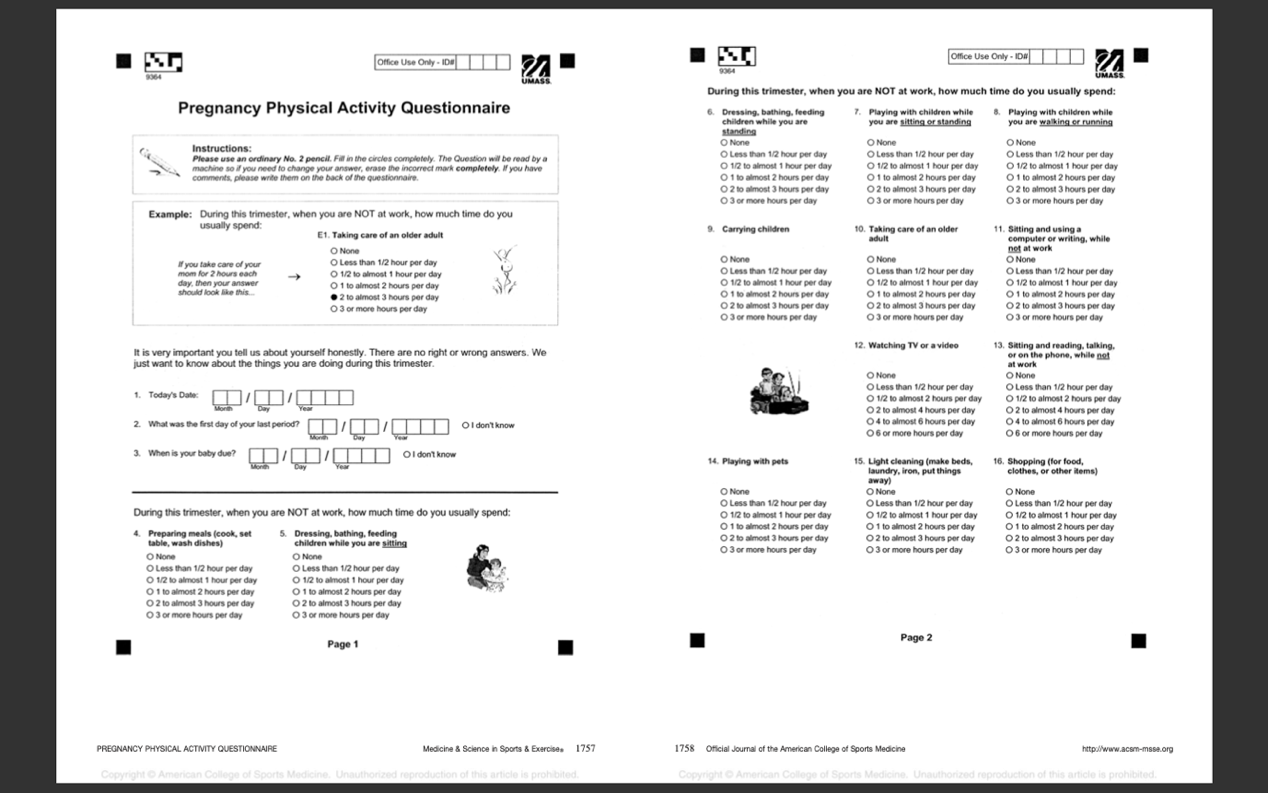
**

**
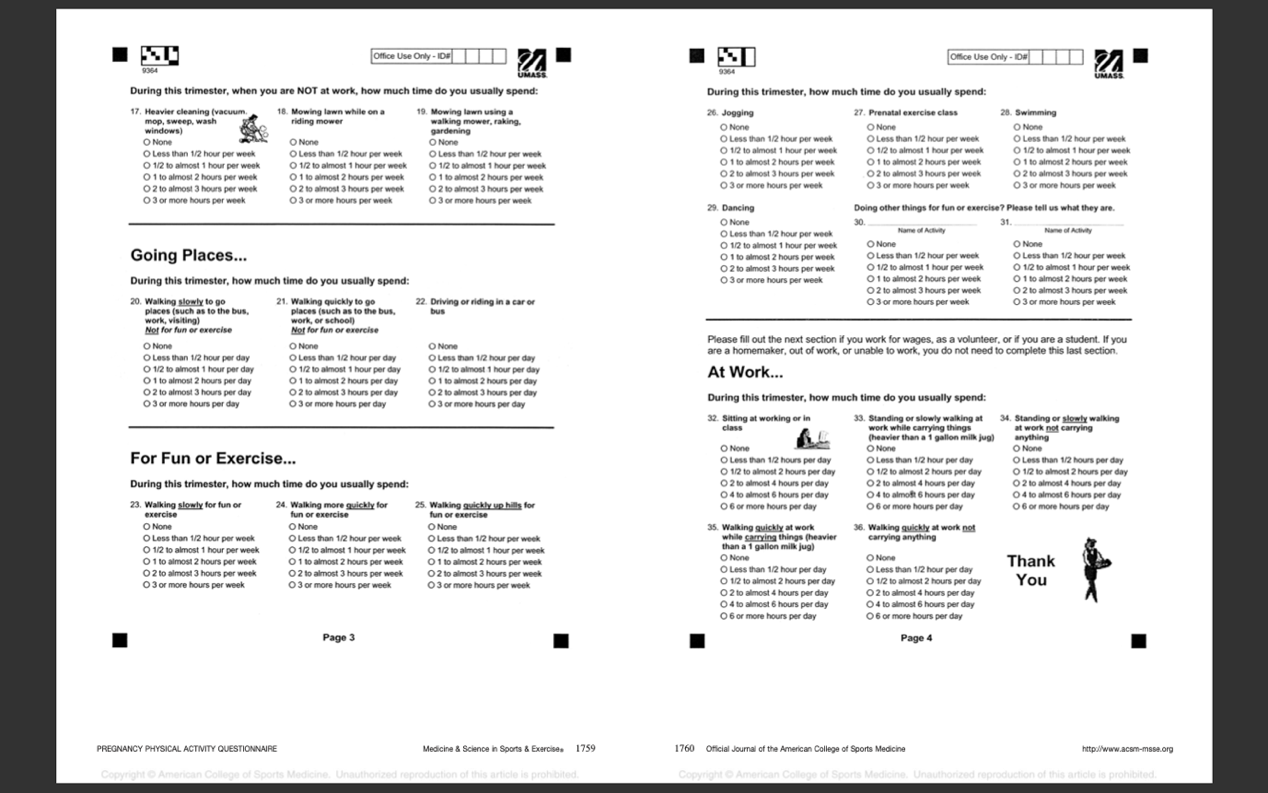
**

Supplementary File 2: Dietary Nutrient Intake Summary Chart

|  |  |  | n=23(%) | | Control group  (n=12)(%) | | Probiotic group  (n=11)(%) | | Control group | | Probiotic group | |  |
| --- | --- | --- | --- | --- | --- | --- | --- | --- | --- | --- | --- | --- | --- |
| Nutrition | Recommended Nutrient Intake | UL  Tolerable Upper Intake | Reach RNI(cases,%) | Below standards RNI(cases,%) | Reach RNI(cases,%) | Below standards RNI(cases,%) | Reach RNI(cases,%) | Below standards RNI(cases,%) | Mean | Mean | | *p* value | |
| Kcal | 2250 |  | 18 (78.26%) | 5  (21.74%) | 9  (75%) | 3  (25%) | 9  (81.82%) | 2 (18.18%) | 2415.48 ± 543.18 | 2488.83 ± 350.88 | | 0.70 | |
| Protein | RNI 85g |  | 21 (91.30%) | 2  (8.70%) | 10  (83.33%) | 2  (16.67%) | 11  (100%) | 0  (0%) | 122.26 ± 38.75 | 153.35 ± 47.13 | | 0.09 | |
| Fat^2^ | 20-30% of energy (50~75g) |  | 4(17.39%)  (18cases, 78.26% exceed standard) | 1  (4.35%) | 1 (8.33%) (10cases, 83.34% exceed standard) | 1  (8.33%) | 3 (27.27%) (8cases,  72.73%  exceed standard) | 0  (0%) | 90.76 ± 24.14 | 95.68 ± 26.87 | | 0.64 | |
| Carbohydrate | EAR 130g |  | 23  (100%) | 0  (0%) | 12  (100%) | 0  (0%) | 11  (100%) | 0  (0%) | 291.61 ± 86.13 | 267.12 ± 44.18 | | 0.40 | |
| Fiber |  |  | 23  (100%) | 0  (0%) | 12  (100%) | 0  (0%) | 11  (100%) | 0  (0%) | 14.22 ± 7.40 | 13.92 ± 4.12 | | 0.90 | |
| SFA^1^ | ﹤10% of energy (25g) |  | 9  (39.13%) | 14  (60.87%) | 5  (41.67%) | 7  (58.33%) | 4  (45.45%) | 7  (54.55%) | 25.47 ± 7.31 | 26.60 ± 8.06 | | 0.72 | |
| MUFA |  |  | 23  (100%) | 0  (0%) | 12  (100%) | 0  (0%) | 11  (100%) | 0  (0%) | 31.95 ± 8.99 | 33.06 ± 10.133 | | 0.78 | |
| PUFA |  |  | 23  (100%) | 0  (0%) | 12  (100%) | 0  (0%) | 11  (100%) | 0  (0%) | 17.57 ± 5.16 | 18.78 ± 6.06 | | 0.61 | |
| Vit A | RNI 770ug | UL 2400ug | 18  (78.26%) | 5  (21.74%) | 8  (66.67%) | 4  (33.33%) | 10  (90.91%) | 1  (9.09%) | 1322.95 ± 738.07 | 1552.33 ± 754.25 | | 0.46 | |
| Vit B1 | RNI 1.5mg | UL 50mg | 20  (86.96%) | 3  (13.04%) | 10  (83.33%) | 2  (16.67%) | 10  (90.91%) | 1  (9.09%) | 1.89 ± 0.81 | 2.21 ± 0.74 | | 0.33 | |
| Vit B2 | RNI 1.5mg | UL 50mg | 20  (86.96%) | 3  (13.04%) | 10  (83.33%) | 2  (16.67%) | 10 (90.91%) | 1 (9.09%) | 1.98 ± 0.59 | 2.19 ± 0.46 | | 0.34 | |
| Vit B3 | RNI  12mg |  | 23  (100%) | 0  (0%) | 12  (100%) | 0  (0%) | 11  (100%) | 0  (0%) | 34.72 ± 11.41 | 42.64± 15.41 | | 0.17 | |
| Vit C | RNI 115mg | UL 1000mg | 15  (78.26%) | 8  (34.78%) | 7  (58.33%) | 5  (41.67%) | 8  (72.73%) | 3  (27.27%) | 203.42 ± 134.81 | 191.67 ± 96.09 | | 0.81 | |
| Vit E | AI 14mg | UL 700mg | 21 (91.30%) | 2  (8.70%) | 10  (83.33%) | 2  (16.67%) | 11  (100%) | 0  (0%) | 23.26 ± 8.17 | 24.86 ± 4.95 | | 0.58 | |
| Ca | RNI 1000mg | UL 2000mg | 12  (52.17%) | 11  (47.83%) | 7  (58.33%) | 5  (41.67%) | 5  (45.45%) | 6  (54.55%) | 948.70 ± 384.22 | 927.54 ± 301.84 | | 0.88 | |
| Mg | RNI 370mg | UL 700mg | 18  (78.26%) | 5  (21.74%) | 9  (75%) | 3  (25%) | 9  (81.82%) | 2  (18,18%) | 456.66 ± 156.71 | 471.39 ± 77.04 | | 0.78 | |
| Fe | RNI 29mg | UL 50mg | 15  (65.22%) | 8  (34.78%) | 7  (58.33%) | 5  (41.67%) | 8  (72.73%) | 3  (27.27%) | 29.60 ± 8.11 | 33.08 ± 8.17 | | 0.31 | |
| Zn | RNI 9.5mg | UL 35mg | 23  (100%) | 0  (0%) | 12  (100%) | 0  (0%) | 11  (100%) | 0  (0%) | 21.45 ± 5.99 | 25.43 ± 9.58 | | 0.24 | |
| Se | RNI 65ug | UL 400ug | 21 (91.30%) | 2  (8.70%) | 11  (91.67%) | 1  (8.33%) | 10 (90.91%) | 1 (9.09%) | 97.55 ± 28.18 | 133.96 ± 49.55 | | 0.05 | |
| 注: SFA: Saturated Fatty Acids、MUFA: Monounsaturated Fatty Acids、PUFA: Polyunsaturated Fatty Acids、RNI:Recommended Nutrient Intake、AI: Adequate Intake、UL: Tolerable Upper Intake Levels、EAR: Estimated Average Requirement  1：SFA reaching RNI means the intake is less than 25g, and not reaching RNI means the intake is greater than 25g 2：Fat reaching RNI means that the intake is 50 ~ 75g, exceeding the standard means that the intake is greater than 75g, and not reaching RNI means that the intake is less than 50g | | | | | | | | | | | | | |
